# Scabies and Bacterial Superinfection among American Samoan Children, 2011–2012

**DOI:** 10.1371/journal.pone.0139336

**Published:** 2015-10-12

**Authors:** Laura Edison, Amanda Beaudoin, Lucy Goh, Camille E. Introcaso, Diana Martin, Christine Dubray, James Marrone, Chris Van Beneden

**Affiliations:** 1 Epidemiology Workforce Branch, Centers for Disease Control and Prevention, Atlanta, Georgia, United States of America; 2 Lyndon B. Johnson Tropical Medical Center, Department of Pediatrics, Pago Pago, American Samoa; 3 Pennsylvania Center for Dermatology, Philadelphia, Pennsylvania, United States of America; 4 Division of Parasitic Diseases and Malaria, Centers for Disease Control and Prevention, Atlanta, Georgia, United States of America; 5 Respiratory Diseases Branch, Centers for Disease Control and Prevention, Atlanta, Georgia, United States of America; The Australian National University, AUSTRALIA

## Abstract

**Background:**

Scabies, a highly pruritic and contagious mite infestation of the skin, is endemic among tropical regions and causes a substantial proportion of skin disease among lower-income countries. Delayed treatment can lead to bacterial superinfection, and treatment of close contacts is necessary to prevent reinfestation. We describe scabies incidence and superinfection among children in American Samoa (AS) to support scabies control recommendations.

**Methodology/Principal Findings:**

We reviewed 2011–2012 pharmacy records from the only AS pharmacy to identify children aged ≤14 years with filled prescriptions for permethrin, the only scabicide available in AS. Medical records of identified children were reviewed for physician-diagnosed scabies during January 1, 2011–December 31, 2012. We calculated scabies incidence, bacterial superinfection prevalence, and reinfestation prevalence during 14–365 days after first diagnosis. We used log binomial regression to calculate incidence ratios for scabies by age, sex, and county. Medical record review identified 1,139 children with scabies (incidence 29.3/1,000 children aged ≤14 years); 604 (53%) had a bacterial superinfection. Of 613 children who received a scabies diagnosis during 2011, 94 (15.3%) had one or more reinfestation. Scabies incidence varied significantly among the nine counties (range 14.8–48.9/1,000 children). Children aged <1 year had the highest incidence (99.9/1,000 children). Children aged 0–4 years were 4.9 times more likely and those aged 5–9 years were 2.2 times more likely to have received a scabies diagnosis than children aged 10–14 years.

**Conclusions/Significance:**

Scabies and its sequelae cause substantial morbidity among AS children. Bacterial superinfection prevalence and frequent reinfestations highlight the importance of diagnosing scabies and early treatment of patients and close contacts. Investigating why certain AS counties have a lower scabies incidence might help guide recommendations for improving scabies control among counties with a higher incidence. We recommend interventions targeting infants and young children who have frequent close family contact.

## Introduction

Scabies is an intensely pruritic, highly contagious skin infestation caused by the arachnid mite *Sarcoptes scabiei* variant *hominus*. Scabies was added to the World Health Organization’s list of neglected tropical diseases (http://www.who.int/neglected_diseases/diseases/scabies/en/) in November 2013 because it accounts for a substantial proportion of skin disease among low-income countries, and despite the substantial burden of disease, it has been largely absent from the global health agenda [1,2]. The addition of scabies to this list increases publicity for this largely ignored disease and recognizes that new programs are needed to control scabies and its sequelae [1,2]. The highest rates of scabies are reported among children from low-income countries where the prevalence typically ranges from 0.2% to 24%; however, it is endemic among tropical regions, with prevalence ranging from 10% to 25%, and areas in the Pacific region have reported prevalence as high as 50% [1,3–11]. Although the incidence of scabies has not been determined previously in American Samoa, a 2009 study of 8,767 children in western Samoa reported a prevalence of 4.3%, whereas a prospective study in Fiji reported a prevalence of 18% among school children (aged 5–15 years) and 14% among infants [9,10].

Scabies transmission occurs through direct and prolonged contact, and possibly through sharing contaminated clothing or bedding. Infestation causes intense pruritus, particularly at night, often causing sleep disruption. Excoriation of lesions can lead to secondary bacterial superinfections [4,12,13], therefore treating an infestation early during the disease process may prevent bacterial superinfection and scabies transmission to close contacts, and treating scabies in a community leads to a concurrent reduction in rates or pyoderma [10,14]. The most common causes of bacterial skin and superinfection among low-income nations are group A *Streptococcus* (GAS) and *Staphylococcus aureus* [3,9,14–16]. GAS skin infections can lead to poststreptococcal glomerulonephritis, and certain epidemiologists hypothesize that acute rheumatic fever might also be a sequela [6,9,14,17]. Reinfestations can be prevented by properly applied permethrin cream (5%), or other effective scabicidal treatment, administered to all close contacts in addition to the patient and environmental remediation, including washing contaminated bedding and clothing in hot water [1,4,12,13,18]. In addition to sleep disturbance, consequences of scabies include loss of school or work, economic burden on families or persons, and secondary bacterial superinfection [4,9,12,13]. Risk factors for scabies among low-income countries include young age, illiteracy, low socioeconomic status, shared clothing and beds, and household overcrowding [4,6–8,11,19,20].

Scabies among American Samoan (AS) children is perceived to be a substantial problem by the local medical community, with certain patients reportedly presenting for care multiple times because of persistent infestation, reinfestation, and bacterial superinfection. The incidence of scabies had not been described in AS; a fuller understanding of the burden of scabies is imperative for developing targeted community interventions. We conducted a retrospective study to describe the estimated incidence of scabies infestation and the prevalence of reinfestation and bacterial superinfection of scabies lesions among infants and children aged ≤14 years in AS; we achieved these goals and made recommendations to control the epidemic on the basis of our report.

## Methods

### Study Area

AS is a U.S. territory located in the South Pacific, with an estimated population of 55,500 persons (median age 22 years; average life expectancy 75 years), a land area of 76.1 square miles, and a tropical climate [21,22]. The majority of residents reside on Tutuila Island, and a limited number of persons reside on outlying islands. The island of Tutuila is divided into nine counties. The per capita gross domestic product is approximately $8,000/year, and the infant mortality rate is 9.15 deaths/1,000 live births. Ninety-three percent of the population resides in urban areas, and 97% in homes with an average household size of 5.6 persons [21,22]. The Lyndon B. Johnson Tropical Medical Center (LBJTMC), the only hospital, includes a pharmacy and outpatient clinic; four other outpatient clinics are located in AS. All health care facilities are located on Tutuila Island and utilize a common electronic medical record system. Patients treated at the hospital and all outpatient clinics obtain prescription medications from the LBJTMC pharmacy, the only pharmaceutical dispensary on the island. Permethrin cream (5%) is the only prescription scabicide treatment available in AS. Medical care is provided for all pediatric patients at a cost to the family of ≤$20/visit, including prescribed medication.

### Case Definition and Ascertainment

We defined a case of scabies as physician-diagnosed infection with *Sarcoptes scabiei* variant *hominus* in a patient aged ≤14 years, during January 1, 2011–December 31, 2012. Scabies diagnosis in AS is based on clinical presentation and distribution of lesions, presence of symptoms (particularly intense itching that is worse at night or irritability and sleep disturbances among infants), and often a history of close contact with a person with a pruritic rash. Reinfestation was defined as a scabies diagnosis during 14–365 days from a previous scabies diagnosis; we used 14 days as a minimum to avoid counting return visits for the initial infestation, and 365 days as a maximum to ensure all records were followed for the same period postinfestation. Bacterial superinfection of scabies lesions was defined as one or more of the following: physician-diagnosed superinfection, a skin infection in the same location as the scabies lesions at the time of scabies diagnosis, or antibiotic prescription at the time of scabies diagnosis, with no other indication for antibiotics noted in the medical record.

To determine the estimated incidence of scabies in AS we queried pharmacy records in August 2013 to obtain a list of all permethrin prescriptions filled during January 1, 2011–December 31, 2012. We reviewed medical records for patients who were aged ≤14 years at the time the prescription was filled. Data were abstracted into a Microsoft^®^ Access® (Microsoft Corporation, Redmond, Washington) database for all children who met the case definition. Data collected included demographics, date of all scabies diagnoses, and presence of bacterial superinfection. All personally identifiable information was removed by the authors from the dataset prior to analysis (S1 Dataset). This study was reviewed by the Centers for Disease Control and Prevention for human subjects protection and was determined to be an emergent public health response and was therefore not considered human subjects research and IRB approval was not required.

### Analysis

We calculated the estimated average annual incidence of scabies (scabies cases/1,000 children) by age, age category, sex, and county of residence by using population estimates in the denominator obtained from the 2010 U.S. Census [21]. We used log binomial regression to calculate incidence ratios for scabies and 95% confidence intervals by sex (using females as the reference group), age category (10–14 years as the referent), and county (Sa’ole as the referent). Reference groups selected were based on the lowest scabies incidence. County of residence was determined on the basis of village indicated in the medical record, and those with missing village or not from Tutuila Island were not counted in the analysis that included county. All variables were included in the model, and we assessed for interaction and confounding. We calculated the prevalence of reinfestation among children with scabies during 2011 and mean time between scabies diagnoses. All calculations were performed by using Microsoft^®^ Excel^®^ (Microsoft Corporation, Redmond, Washington) or SAS^®^ version 9.3 (SAS Institute, Inc., Cary, North Carolina). ArcMap 10.2 (Redlands, CA) was used to create a county-level average annual scabies incidence rate map using a freely available vector shapefile downloaded from the AS Dept. of Commerce website (http://doc.as.gov/).

## Results

Pharmacy record review identified 1,733 children aged ≤14 years with a prescription for permethrin cream (5%) during 2011–2012; 1,139 of these children had a diagnosis of scabies in their medical record. Among the AS population of 19,425 children aged ≤14 years, we identified 613 children with scabies during 2011 and 526 during 2012. The annual average was 570 cases, or 29.3 cases/1,000 children. Estimated incidence was highest among children aged 0–4 years and declined with advancing age; children aged <1 year had the highest incidence (99.9/1,000 children), and children aged 14 years had the lowest incidence (7.3/1,000 children) (Table 1). Compared to children aged 10–14 years, children aged 0–4 years were 4.9 times more likely to have been diagnosed with scabies, and children aged 5–9 years were 2.2 times more likely to have been diagnosed with scabies (Table 2).

**Table 1:** Scabies* and bacterial superinfection among American Samoan children, 2011–2012.

Age^†^ (yrs)	No. of children^‡^	No. of scabies cases	Annual incidence /1,000 children**	No. of super-infected cases^††^ (%)
0	1,477	295	99.9	135 (45.8)
1	1,295	163	62.9	85 (52.1)
2	1,210	80	33.1	51 (63.8)
3	1,249	82	32.8	54 (65.9)
4	1,380	79	28.6	42 (53.2)
5	1,369	63	23.0	42 (66.7)
6	1,356	69	25.4	43 (62.3)
7	1,238	59	23.8	31 (52.5)
8	1,282	59	23.0	31 (52.5)
9	1,290	55	21.3	25 (45.5)
10	1,303	44	16.9	22 (50.0)
11	1,266	26	10.3	12 (46.2)
12	1,224	22	9.0	12 (54.5)
13	1,250	25	10.0	11 (44.0)
14	1,236	18	7.3	8 (44.4)
Total	19,425	1,139	29.3	604 (53.0)

*Physician-diagnosed scabies cases among patients aged ≤14 years at the time of diagnosis, during January 1, 2011─December 31, 2012. Children had received one or more scabies diagnoses.

^†^Age at which diagnosis of scabies was received.

^‡^American Samoan population from the 2010 US Census [21].

**Average annual incidence of scabies during 2011 and 2012.

^††^Among all children with scabies during 2011–2012.

**Table 2:** Scabies* among American Samoan children aged 0–14 years by age category, sex, and county, 2011–2012.

	No. of children^†^	No. of scabies cases	Annual incidence /1,000 children^‡^	Incidence ratio**	95% CI
**Age (yrs)** ^**††**^					
0–4	6,611	699	52.9	4.9	4.1–5.9
5–9	6,535	305	23.3	2.2	1.8–2.7
10–14	6,279	135	10.8	Ref.	
**Sex**					
Male	9.,324	660	32.7	1.3	1.1–1.4
Female	10,101	479	25.7	Ref.	
Total	19,425	1,139	29.3		
**County** ^**‡‡**^					
Ituau	1,568	127	40.5	3.2	2.0–5.0
Maoputasi	3,545	281	39.6	3.1	2.0–4.8
Leasina	634	48	37.9	2.9	1.8–4.8
Lealataua	1,757	114	32.4	2.5	1.6–4.0
Sua	1,058	63	29.8	2.3	1.4–3.7
Tualauta	7,419	364	24.5	1.9	1.2–2.9
Vaifanua	866	41	23.7	1.8	1.1–3.1
Tualatai	1,322	60	22.7	1.8	1.1–2.9
Sa'ole	811	21	12.9	Ref.	
Total	1,568	1,119	29.5		

CI = confidence interval.

*Physician-diagnosed scabies among patients aged ≤14 years at the time of diagnosis, during January 1, 2011─December 31, 2012. Children had ≥1 scabies diagnosis.

^†^American Samoan population from the 2010 US Census Bureau report [21].

^‡^Average annual incidence of scabies during 2011 and 2012.

**Ratio of incidence of specified group compared with reference group.

^††^Age at which diagnosis of scabies was received.

^‡‡^Only includes children with known village who reside on Tutuila Island.

**Fig 1:**
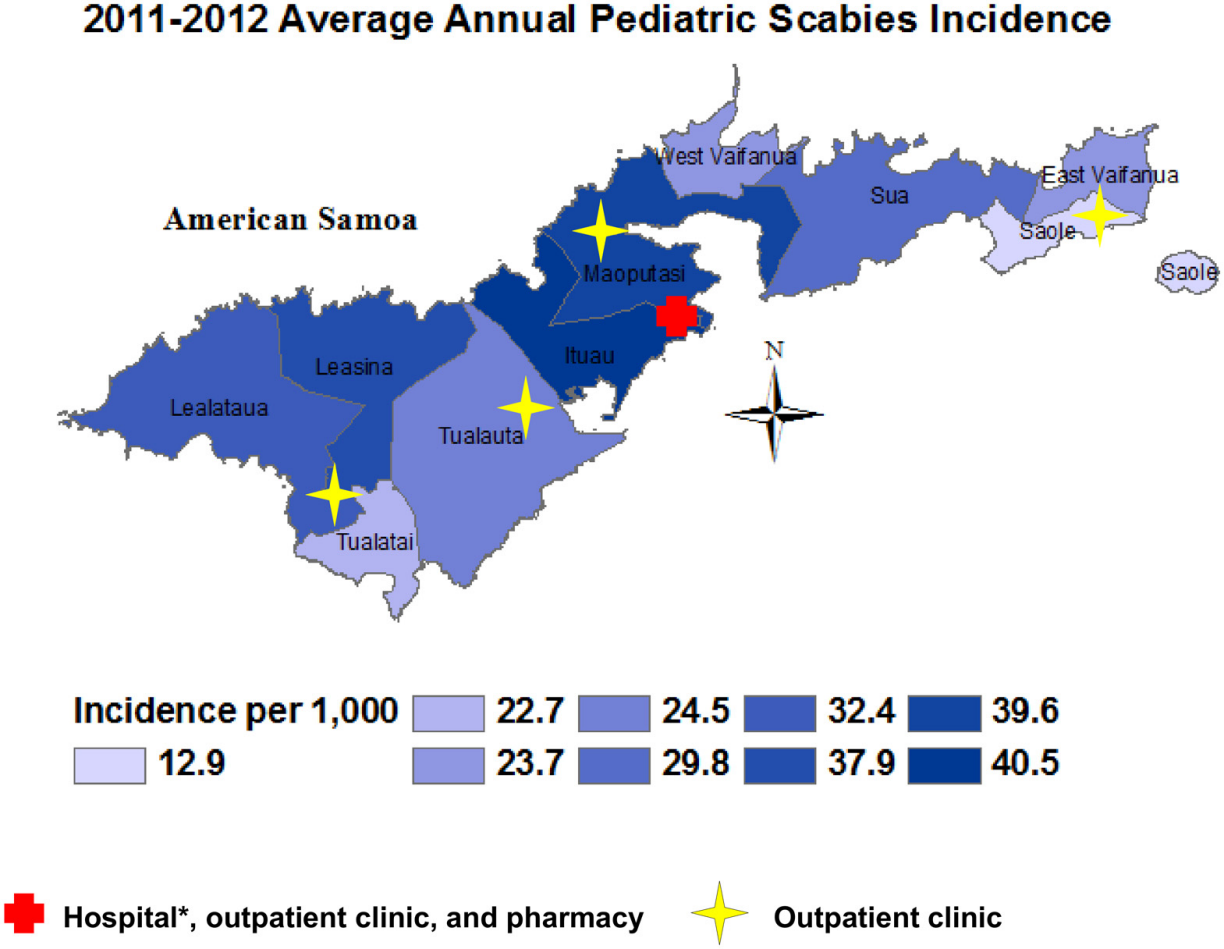
Average annual pediatric scabies incidence by county, American Samoa.

Incidence also varied by county; children who reside in each county on Tutuila Island besides Sa’ole were significantly more likely to have scabies than those living in Sa’ole County (incidence ratio range 1.8–3.2) (Table 2 and Fig 1). None of the variables tested were substantial effect modifiers or confounders.

**Fig 2:**
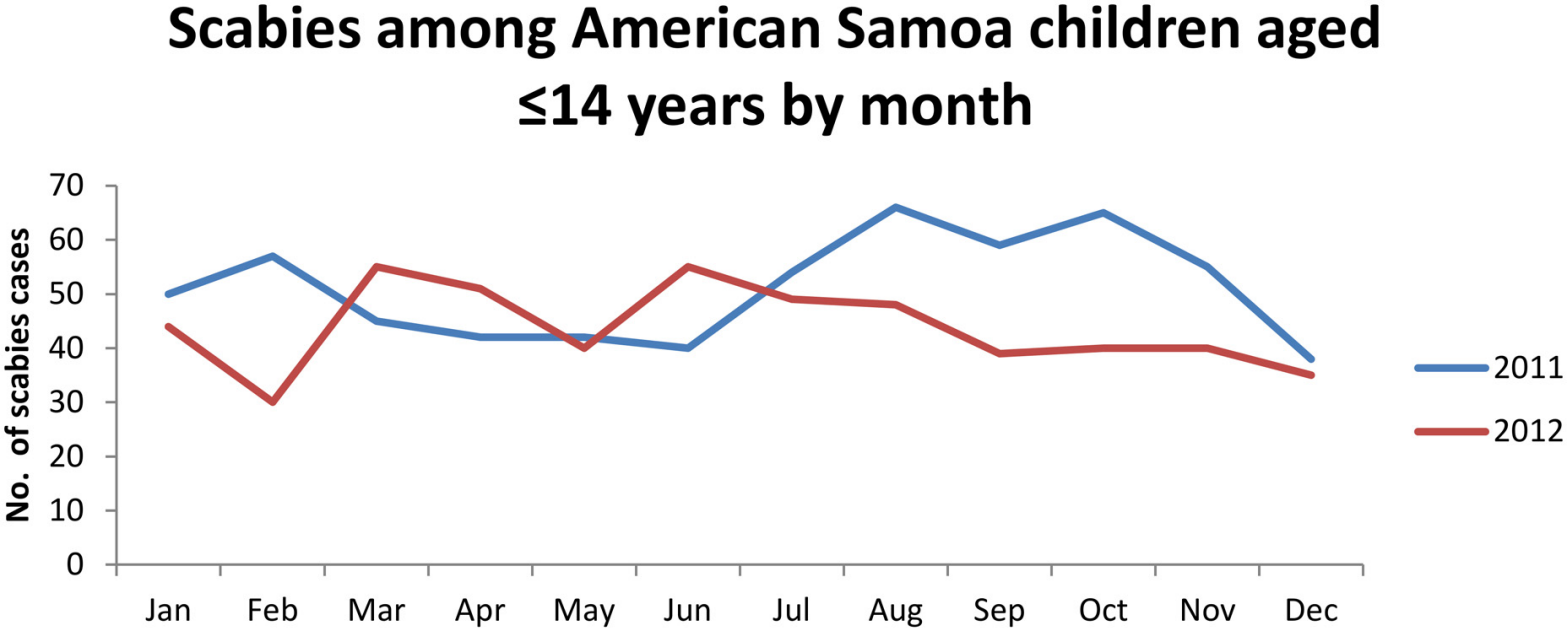
Scabies incidence by month among American Samoan children aged ≤14 years.

Bacterial superinfection was present at the time of scabies diagnosis among 604/1,139 (53.0%) children (Table 1). Among 613 children with scabies during 2011, 94 (15.3%) had one or more reinfestation; 64 (68.1%) of these were superinfected at the time of the second scabies diagnosis. Antibiotics prescribed included cephalexin, amoxicillin, penicillin, azithromycin, and trimethoprim-sulfmethoxazole. The median time between the first and second diagnosis was 86 days (range 14–341 days). Scabies incidence did not vary consistently by month of diagnosis (Fig 2).

## Discussion

Our study demonstrates that scabies and associated bacterial superinfections cause substantial morbidity among AS children. During 2011–2012, an average of 29.3/1,000 children and approximately 1 of 10 infants received a scabies diagnosis each year. Scabies incidence decreased with increasing age, which is consistent with previous studies [1,13]. This likely results from younger children being in close contact with multiple family members and often being held, which provides opportunities for exposure to infected family members. The predominance among males contrasts with previous studies; additional research is needed to understand the reasons for this difference [4]. Scabies does not appear to follow a seasonal pattern, which is expected in tropical regions where weather patterns are consistent throughout the year [4,9,23–25].

The difference between the number of children with a permethrin prescription (1,733) and the number of children with a scabies diagnosis (1,139) can be explained by prescribing practices; every household member receives a prescription when a child is diagnosed with scabies, hence many of these children may have been siblings that did not seek medical care.

To understand the variation of scabies incidence by county, we reviewed distances between the pharmacy and outpatient clinics. The pharmacy is located centrally on the island in Maoputasi County, which has among the higher incidences of scabies. However, outpatient clinics are located in counties with both higher and lower incidences (Fig 1), which indicates that distance from outpatient clinics does not influence scabies incidence. In addition, approximately 93% of the population resides in urban areas, indicating that differences between urban and rural lifestyles have limited effect on county differences in scabies incidence [22]. The possibility exists that lifestyle differs by community or that scabies is easily maintained after established locally.

The high frequency of reinfestation and the limited time between recurrent infections indicate that these subsequent infections are likely because of incorrectly applied treatment, treatment failure, or inadequate treatment of the environment or close contacts. This might allow scabies to persist in the child’s environment and among close contacts after being established [26,27].

The high frequency of bacterial superinfection might result from crowded housing and inadequate hygiene [1]. Delays in seeking care also allow time for the secondary lesions to develop in the broken skin. The prevalence of both impetigo and scabies is higher in the South Pacific region than among a majority of low-income nations, and scabies has been identified as a risk factor for bacterial skin infections. For example, in Fiji, children with scabies were 2.4 times more likely to have impetigo than children without scabies [4,6,9,10,28]. Additionally, intervention studies designed to treat scabies have reported a substantial reduction in pyoderma even without use of antibiotics or other pyoderma-specific interventions [16,29–31]. Bacterial superinfection can lead to serious sequelae, including cellulitis and abscesses. Among more severe cases, bacteremia and sepsis can occur [14].

Our study has likely underestimated the incidence of scabies among AS children because we only included children who sought medical care at LBJTMC or one of the outpatient clinics and filled a prescription of permethrin, and we might have missed cases among persons not seeking treatment by using traditional or over-the-counter medicine obtained outside the medical system, not filling prescriptions because of expense or pharmacy inaccessibility, or obtaining medication from a friend or family member. All household contacts are prescribed permethrin when scabies is diagnosed, yet only those children who were treated by a doctor and had received a scabies diagnosis were included in this analysis. However, since scabies is diagnosed clinically in AS, it is possible some initial scabies diagnoses and reinfestations were actually misdiagnosed since scabies can mimic many common skin diseases in the tropics. Another limitation is our use of a broad definition of bacterial superinfection, potentially inflating this estimate; cases of unrelated bacterial infection might be included if not clearly documented in the record.

Although scabies is known to be particularly prevalent throughout the Pacific region, medical personnel report socioeconomic and lifestyle concerns that are particular to AS and might contribute to the problem. Traditional medicine is commonly the first choice for care; families often do not seek care at the hospital or outpatient clinic until an infestation, or secondary infection, is severe, allowing time for scabies to spread to other family members. Living conditions are often crowded, with certain families sharing a living space, beds, and clothing. Because permethrin prescriptions cost $10/patient, treating a multiple family members might not be feasible for multiple families. Finally, AS families often do not have access to hot water to wash clothing, and do not have enough bedding and clothing to bag everything for seven days to kill the mites. This can allow scabies mites to survive in the environment, infesting family members, and leading to reinfestations. (Personal communication with Lucy Goh and James Marrone.)

Our study describes the previously unknown incidence of scabies among AS children and indicates that age and county are associated with scabies incidence. Additional community-based studies are needed to examine additional risk factors for scabies. Such studies might include cross-sectional and prospective studies of children in school or other community venues. This might allow a more accurate assessment of the incidence of scabies, regardless of whether the children present for medical care. Parent or child interviews might help elucidate risk factors for and knowledge and attitudes about scabies. Understanding county differences in lifestyle, knowledge, attitude, and behavior concerning scabies might support recommendations for scabies control and help target interventions to areas with higher incidence.

On the basis of the success of previous education efforts in different community settings, we recommended education for medical providers about scabies diagnosis and creation of recommended protocols for scabies treatment and environmental control [1,9,11]. Existing algorithms used to diagnose common childhood skin conditions are available, which can be useful for training providers [32]. We propose that interventions should focus on families with young children and on those families residing in counties with the highest incidence. Education about the importance of early treatment to prevent secondary infection and treatment of all close contacts and the environment to prevent reinfestation and transmission should be emphasized. Mass drug administration campaigns, by using either permethrin cream (5%) or oral ivermectin, have led to temporary decreases in the incidence of scabies among certain countries and might be considered for AS. However, these interventions are costly and often impractical, given the limited resources. Timely diagnosis, treatment, increased awareness and case finding, and education for health care workers, patients, and the community are the mainstays of scabies prevention [1,4,9,11,12,29–31].

## Supporting Information

S1 Dataset Dataset containing deindentified data abstracted from medical records of American Samoan children diagnosed with scabies during 2011–2012. (XLSX)
